# Textile-Based Adsorption Sensor via Mixed Solvent Dyeing with Aggregation-Induced Emission Dyes

**DOI:** 10.3390/ma17081745

**Published:** 2024-04-11

**Authors:** Seong Gyun Hong, Byeong M. Oh, Jong H. Kim, Jea Uk Lee

**Affiliations:** 1 Department of Advanced Materials Engineering for Information and Electronics, Integrated Education Institute for Frontier Science and Technology (BK21 Four), Kyung Hee University, 1732 De-ogyeong-daero, Giheung-gu, Yongin-si 17104, Gyeonggi-do, Republic of Korea; tjdrbs828@khu.ac.kr; 2 Department of Molecular Science and Technology, Ajou University, 206, World Cup-ro, Yeongtong-gu, Suwon-si 16499, Gyeonggi-do, Republic of Korea; hanmir980@ajou.ac.kr (B.M.O.); jonghkim@ajou.ac.kr (J.H.K.)

**Keywords:** aggregation-induced emission dyes, recycled fabric, solvent dyeing, smart textile, sensor

## Abstract

This study demonstrates a novel methodology for developing a textile-based adsorption sensor via mixed solvent dyeing with aggregation-induced emission (AIE) dyes on recycled fabrics. AIE dyes were incorporated into the fabrics using a mixed solvent dyeing method with a co-solvent mixture of H_2_O and organic solvents. This method imparted unique fluorescence properties to fabrics, altering fluorescence intensity or wavelength based on whether the AIE dye molecules were in an isolated or aggregated state on the fabrics. The precise control of the H_2_O fraction to organic solvent during dyeing was crucial for influencing fluorescence intensity and sensing characteristics. These dyed fabrics exhibited reactive thermochromic and vaporchromic properties, with changes in fluorescence intensity corresponding to variations in temperature and exposure to volatile organic solvents (VOCs). Their superior characteristics, including a repetitive fluorescence switching property and resistance to photo-bleaching, enhance their practicality across various applications. Consequently, the smart fabrics dyed with AIE dye not only find applications in clothing and fashion design but demonstrate versatility in various fields, extending to sensing temperature, humidity, and hazardous chemicals.

## 1. Introduction

Traditional organic luminescent materials manifest notable fluorescence in diluted solutions [1,2]. However, their luminescent properties tend to diminish or completely disappear when encountered in high-concentration solutions or solid states due to the aggregation-caused quenching (ACQ) effect [3,4,5]. This phenomenon impacts fluorescent substances, such as triazole derivatives, rendering them impractical in their solid form [6]. In 2001, Tang et al. discovered a series of silole derivatives that remain non-emitting in dilute solutions but exhibit exceptional fluorescence when aggregated in high-concentration solutions or in solid form, coining the term ‘aggregation-induced emission’ (AIE) [7,8,9]. Restricted intramolecular rotation plays a pivotal role in the aggregation and release of AIE [10,11]. AIE luminescent materials, such as high-efficiency tetraphenylethylene (TPE), confer advantages over conventional ACQ materials, including a high signal-to-noise ratio, improved sensitivity, and strong resistance to photobleaching [12,13]. AIE materials have been utilized in a variety of applications, including biological imaging, biosensors, optoelectronic devices, chemical sensors, fiber optic communications, and displays [14].

Attempts to introduce AIE dyes into textiles have been reported in a variety of ways [15,16]. Ding and colleagues successfully implemented a one-step dry–wet spinning method to fabricate luminescent fibers, incorporating AIE agents and polydimethylsiloxanes [17]. The color spectrum of the luminescent fiber has been successfully tuned through the modification of the AIE agent (AIEgen) structure, enabling the display of diverse monochromatic hues, including red, green, and blue colors. Zhao et al. developed a flexible, multi-functional, Janus-structured nanofiber that emitted white light through the electrospinning of AIEgens-doped thermoplastic polyurethane [18,19]. Zhang et al. developed a cross-linked fluorescent porous polymer film composed of polyhedral oligomeric vinylsilsesquioxane moieties and TPE units through a thiol-ene “click” reaction [20]. This film exhibited a well-organized porous structure due to the presence of polyhedral oligomeric vinylsilsesquioxane moieties while simultaneously displaying strong fluorescence from TPE with the AIE property. Despite ongoing efforts to integrate AIE materials with fibers using diverse approaches, the choice of materials and applications employed in these studies are still limited and predominantly confined to TPE derivatives [21,22,23,24,25].

As global awareness of environmental issues continues to grow, the reuse of polymer materials is becoming increasingly necessary [26]. In particular, known for its versatility and recyclability, polyester has emerged as a staple material in a variety of industries. Unlike many other polymer materials, polyester can be easily collected and repurposed into new products, demonstrating its potential for circularity. Accordingly, research on developing new materials by utilizing recycled polyester has been reported [27]. On the other hand, despite its high usage, research on recycled polyester fiber has rarely been conducted. Unlike polyester plastics, the recycled polyester fiber maintains performance and durability close to conventional products, making it an excellent framework for research on various flexible devices, such as textile-based sensors and electronic devices [28,29]. 

**Figure 1:**
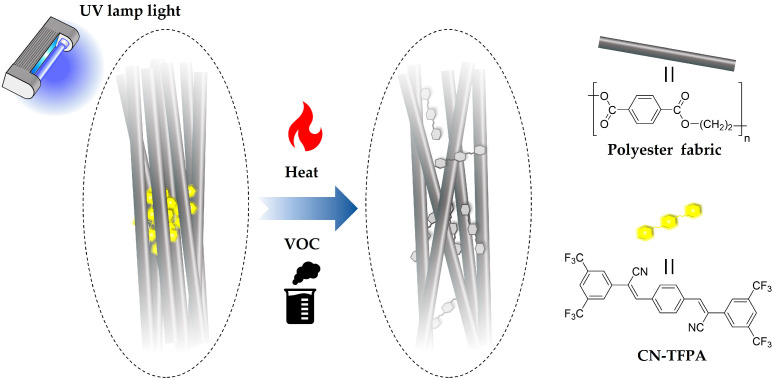
Schematic diagram of the sensing mechanism of AIE-dyed fabric.

In this study, we present a novel approach to develop a textile-based adsorption sensor by manipulating the physical properties of AIE dye through the combination of H_2_O and organic solvents as the dyeing co-solvent. As the AIE dye, the π-conjugated dicyanodistyrylbenzene-based derivative, (2Z,2′Z)-3,3′-(1,4-phenylene)bis(2-(3,5-bis-(trifluoromethyl)phenyl)acrylonitrile) (CN-TFPA) was utilized [30]. The CN-TFPA dye is a material that tends to self-assemble into highly ordered crystalline structures and exhibits an AIE phenomenon instead of the aggregation-caused fluorescence quenching that is normally found for π-electron molecules. Utilizing the distinct characteristics and photoluminescence (PL) mechanism of CN-TFPA dye, the recycled fabric was dyed using a mixed solvent dyeing method. The fluorescence properties and the microstructure of the CN-TFPA dye in both the solution phase and the fabric dyeing state were analyzed using a fluorescence spectrometer and scanning electron microscope, respectively. As a result, the AIE-dyed fabrics demonstrated responsive thermochromic and vaporchromic properties, showcasing potential as smart textile sensors (Figure 1). 

## 2. Materials and Methods

### 2.1. Materials

N,N-dimethylformamide (DMF, 99.8%, Samchun Chemicals, Republic of Korea) and tetrahydrofuran (THF, 99.8%, Samchun Chemicals Co., Ltd., Republic of Korea) were employed for dyeing and vapor sensing. Sodium hydroxide (97.0%, Sigma-Aldrich, USA) and sodium hydrosulfite (82%, Sigma-Aldrich, USA) were applied for reduction clearing. Terephthalaldehyde (99%, Sigma-Aldrich, USA) and 2-(3,5-bis(trifluoromethyl)phenyl)acetonitrile (98%, Sigma-Aldrich, USA) were used for synthesis. t-Butyl alcohol (Sigma-Aldrich, USA), dichloromethane (99.8%, Sigma-Aldrich, USA), and methanol (Sigma-Aldrich, USA) were used as solvents. Tetrabutylammonium hydroxide (Sigma-Aldrich, USA) was used as a catalyst. All chemicals were used without further purification. The recycled polyester fabric (KOHASID, Republic of Korea) was purified with power gel detergent (Persil, Republic of Korea) at 80 °C for 20 min before use.

### 2.2. Synthesis of AIE Dye

**Figure 2:**
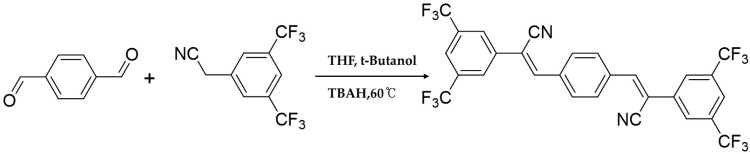
Synthetic procedure of CN-TFPA.

CN-TFPA was synthesized through the previous literature [31]. Briefly, CN-TFPA was synthesized via Knoevenagel condensation between 2-(3,5-bis(trifluoromethyl)phenyl)-acetonitrile and terephthalaldehyde. Terephthaldehyde (0.8 g) and 2-(3,5-bis(trifluoromethyl)phenyl)acetonitrile (3.0 g) were dissolved in 50 mL of t-butyl alcohol (Figure 2). In total, a 0.84 mL aliquot of tetrabutylammonium hydroxide was slowly added to the solution using a reaction temperature of 50 °C and a reaction time of 2 h, and the resultant was purified by filtration and a flash column. For further purification, the product was recrystallized from dichloromethane/methanol solution.

### 2.3. Preparation of AIE-Dyed Fabric

**Figure 3:**
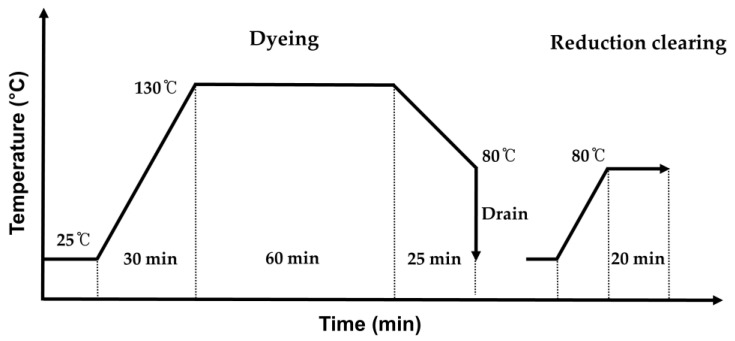
Dyeing process curve utilizing a co-solvent mixture of H_2_O and an organic solvent.

To successfully apply AIE dye on recycled polyester fibers, the agglomeration of CN-TFPA dye was mitigated by employing a co-solvent consisting of H_2_O and DMF. Throughout the experiment, the dyeing effect was evaluated by varying the water ratio from 0% to 80% in the co-solvent. Following the addition of the CN-TFPA dye, the solvent mixture was sonicated for 30 min to prepare a 100 mL solution, resulting in a well-dispersed dye solution. The fabric was introduced into an infrared dyeing machine (DL-6000PLS, DaeLim Starlet, Republic of Korea) with the CN-TFPA solution and was heated gradually from room temperature to 130 °C over a 30 min period. Subsequently, the fabric was dyed at 130 °C for 1 h. The dyed fabric underwent reduction during the cleaning process at 80 °C for 20 min using a solution containing 2 g/L of sodium hydrosulfite and 2 g/L of sodium hydroxide. The overall dyeing process is shown in Figure 3. 

### 2.4. Characterizations

The microstructure of AIE-dyed fabrics was investigated using scanning electron microscopy (SEM, LEO SUPRA 55, Carl Zeiss, Germany). The fluorescence properties of the AIE-dyed fabrics were studied using a fluorescence spectrometer (Quantamaster, HORIBA, Japan) with a xenon lamp as the excitation light source (excitation wavelength of 365 nm, emission wavelength of 470 nm, wavelength range of 400–700 nm) and a fiber optic spectrometer (USB2000+, Ocean Optics, USA) with UV lamp at an emission wavelength of 365 nm. The mechanical properties of the AIE-dyed fabrics were tested with a tensile tester (AGS-X series, Shimadzu, Japan), with gauge lengths and crosshead speeds of 20 cm and 50 mm/min.

## 3. Results and Discussion

### 3.1. Fluorescence Properties of AIE Dye in Solution

**Figure 4:**
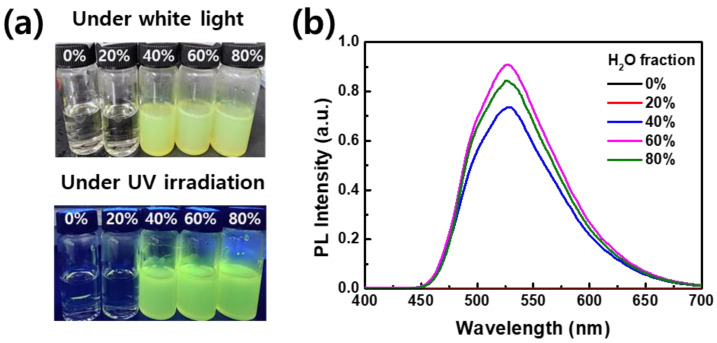
(**a**) Photos of CN-TFPA suspensions at various H_2_O fractions under white light (**top**) and under UV lamp irradiation (**bottom**). The number written on the vial cap indicates the proportion of H_2_O in the solvent mixture. (**b**) PL spectra of CN-TFPA suspension according to the H_2_O fraction.

Conventionally, fluorescent organic nanoparticles have been prepared by introducing an organic surfactant into a dye solution [31]. However, it is important to note that many surfactants have negative effects on the environment. For instance, Nonylphenol Ethoxylates (NPEs) can disrupt the endocrine systems of aquatic organisms, leading to reproductive dysfunction and potentially reducing species diversity and ecosystem health. In this study, a mixed solvent dyeing process was employed using a simple reprecipitation method, which was achieved by controlling the mixing ratio of two types of solvents (with DMF as the good solvent and H_2_O as the non-solvent) without the utilization of the surfactants. The solvent mixture was systematically prepared by gradually adjusting the H_2_O/DMF mixing ratio from 0% to 80%. The CN-TFPA dye was completely soluble in the pure DMF solvent, exhibiting negligible fluorescence under both white light and UV lamp irradiation (Figure 4a). The fluorescence spectrum remained largely unchanged at the H_2_O fraction of 20%. Subsequently, when the water fraction reached 40%, the aggregation of the CN-TFPA dye occurred in the colloidal suspension. Upon aggregation, CN-TFPA emitted bright yellow fluorescence due to restricted intramolecular rotation and vibration. 

Figure 4b depicts the variation in PL spectra with the increasing H_2_O fraction. The fluorescence intensity, which occurred from the 40% H_2_O fraction, obtained its peak value upon reaching 60%. However, at the H_2_O fraction of 80%, a slight decrease in PL intensity was observed. In contrast to the aggregation-caused quenching materials, AIE dyes are known to exhibit an increase in fluorescence as the concentration rises or aggregation occurs. However, some cases have reported that excessive aggregation could result in a modest reduction in PL intensity due to the energy transfer between dye molecules [32]. Nevertheless, the PL intensity at the 80% H_2_O fraction was more than 200 times higher than that emitted from the pure organic solvent (0% H_2_O fraction). These observations underscore the substantial impact of solvent composition on the aggregation-induced emission characteristics of CN-TFPA, demonstrating a promising approach for the fine-tuning of the fluorescence properties of fabrics dyed with CN-TFPA. 

This AIE phenomenon is caused by a synergistic interplay between the planarization of molecules and the head-to-tail J-stacking arrangement of CN-TFPA molecules [29]. Since polar cyano groups are present in the CN-TFPA backbone, the polarity of the molecule increased, leading to an anisotropic electron distribution. This enhances electrostatic interactions between neighboring molecules. Consequently, despite the bulky and repulsive nature of the CF3 end group, the molecule experiences attractive forces. As the molecules move closer to each other, the phenyl rings of the backbone structure undergo planarization to maximize the interaction. However, the repulsion induced by the CF_3_ group causes the approaching molecules to slide along the axis of the molecule, forming the J-aggregation during the self-assembly process. This phenomenon leads to significantly enhanced AIE instead of the aggregation-caused fluorescence quenching that is typically observed for π-electronic molecules.

### 3.2. Fluorescence Properties of Fabrics Dyed with AIE Dye 

**Figure 5:**
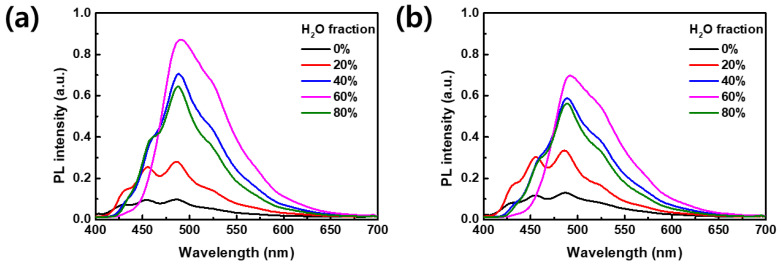
PL spectra of AIE fabrics dyed at concentrations of (**a**) 0.3% o.w.f. and (**b**) 0.5% o.w.f. according to the H_2_O fraction.

Figure 5a shows the fluorescence spectra of the recycled polyester fabrics dyed with the concentration of CN-TFPA at 0.3% on the weight of fiber (o.w.f.). Fluorescence measurements of the dyed fabric samples were conducted in the excitation spectral range of 450 nm to 550 nm, revealing distinct yellow emission characteristics attributed to the presence of CN-TFPA dye. The fabric sample treated with a solution containing 0% of the H_2_O fraction exhibited significantly lower fluorescence intensity. This phenomenon may be attributed to the complete solubility of CN-TFPA in DMF, allowing the CN-TFPA molecules to freely escape from the fabric with the DMF solvent. As the H_2_O fraction gradually increased, CN-TFPA molecules experienced sufficient growth and became trapped within the fibers during the dyeing process, which involved fiber expansion and contraction. Consequently, an increase in the integrated amount of CN-TFPA dye within the fibers corresponded to a notable enhancement in the PL intensity. As with the suspension state, the PL intensity of the dyed fabrics also increased as the H_2_O fraction in the dyeing solvent increased, reaching the highest value at 60%. However, when the H_2_O fraction exceeded 80%, the size of aggregated CN-TFPA dye surpassed the critical threshold for effective penetration into the fibers, resulting in a decrease in PL intensity.

When the dye concentration was increased to 0.5% o.w.f. during the dyeing process, a slight decrease in PL intensity was observed (Figure 5b). This phenomenon may be attributed to the excessive aggregation of the AIE dyes and the resulting energy transfer between them, as previously discussed. Consequently, the recycled polyester fabric was dyed with AIE dye without using additives, such as organic surfactants. Additionally, the fluorescence properties of the dyed fabrics could be controlled by adjusting the solvent mixing ratio.

### 3.3. Microstructural Analysis of AIE Dye and AIE-Dyed Fabrics

**Figure 6:**
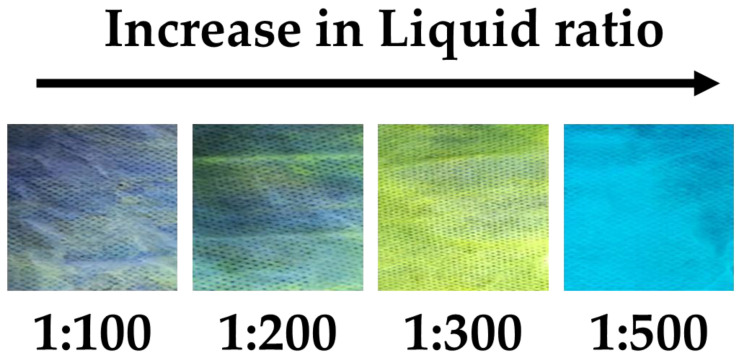
Photos of fabrics dyed at various liquid ratios under UV lamp irradiation.

To explore the optimal mass ratios of fabric-to-solvent (liquid ratios), the experiment was conducted by varying the liquid ratio from 1:100 to 1:500 while keeping the dye concentration and H_2_O fraction fixed at 0.3% and 60%, respectively. Figure 6 displays photo images of the fabrics dyed at different liquid ratios under UV lamp irradiation. When examining the fabrics dyed with liquid ratios of 1:100 and 1:200, it is evident that the fabrics were not uniformly dyed because the solution used at 60% H_2_O fraction did not sufficiently dissolve the CN-TFPA dye. Consequently, the dye became excessively aggregated, hindering its penetration into the fabrics. The fabrics dyed with a liquid ratio of 1:300 exhibited uniform dyeing and displayed strong fluorescence. This seems to be because the three factors of the dye concentration, liquid ratio, and H_2_O fraction were balanced, allowing the appropriate aggregate size of CN-TFPA to penetrate into the fabrics and remain at an optimal size. On the other hand, the fabrics dyed with a liquid ratio of 1:500 exhibited no dyeing effect and displayed the same blue color as the UV lamp. Since the dye concentration was too low to effectively form the dye aggregates, the CN-TFPA dye was not fixed inside the fiber and was completely removed during the washing process. 

**Figure 7:**
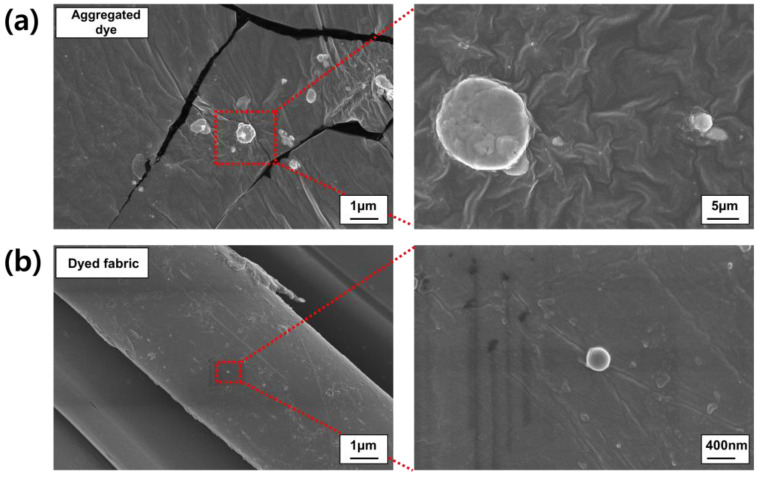
SEM images of the aggregated CN-TFPA dye and the dyed fabric specimen (**a**,**b**).

Aggregated CN-TFPA dye was prepared by completely dissolving CN-TFPA in DMF and, then, adding H_2_O to make the H_2_O fraction 60%. In an undisturbed environment, the CN-TFPA dye aggregates grew from a few to tens of micrometers in size (Figure 7a). On the other hand, no dye aggregates larger than 1.0 μm were found on the AIE-dyed fabric surface (Figure 7b). CN-TFPA molecules, initially in an isolated state, penetrated into the expanded fibers during the dyeing process. Subsequently, during the cooling process, CN-TFPA dye aggregated and grew within the fibers, ultimately becoming confined due to spatial limitation. Following these dyeing processes, CN-TFPA dye was successfully incorporated within the fibers, ensuring robust dyeing and the formation of an adequate size for aggregation-induced emissions.

### 3.4. Sensing Properties of AIE-Dyed Fabrics

**Figure 8:**
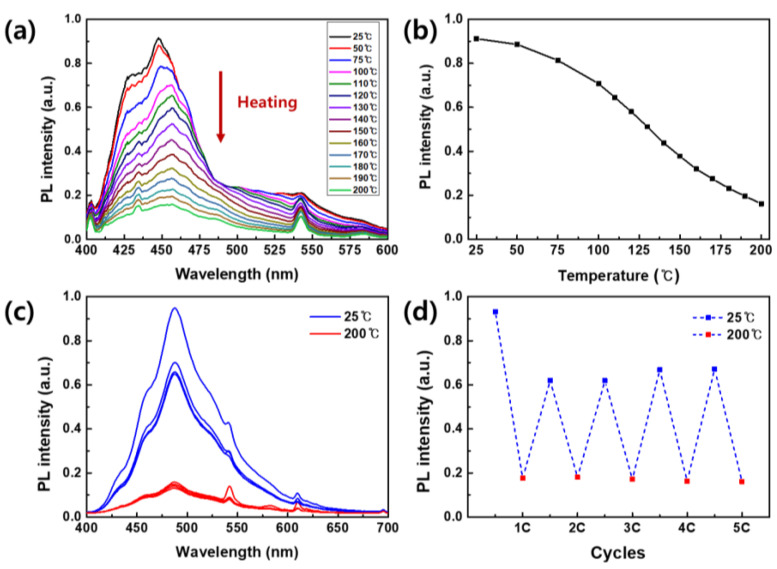
(**a**) PL spectra of AIE-dyed fabrics according to the temperature. (**b**) PL intensity changes at the 450 nm wavelength of AIE fabrics according to the temperature. (**c**) PL spectra of AIE-dyed fabrics according to the repeated heating and cooling at 200 °C and 25 °C. (**d**) PL intensity changes at the 480 nm wavelength of AIE fabrics according to repeated heating and cooling at 200 °C and 25 °C.

Recycled polyester fabrics dyed with CN-TFPA exhibited unique thermochromic properties, allowing for temperature-sensitive fluorescence on/off switching. The PL intensity was measured in real-time as the temperature of the dyed fabric was sequentially raised from 25 to 200 °C (Figure 8a,b). With the rise in temperature, there was a consistent decrease in PL intensity, and the PL intensity at 200 °C was about 80% lower than that at room temperature. To evaluate the reversibility of PL intensity changes with temperature, the AIE-dyed fabrics were subjected to heating at 200 °C and subsequent cooling to 25 °C. The PL intensity was measured over five cycles (Figure 8c,d). While the initial intensity did not fully recover, it displayed highly consistent fluorescent on/off switching characteristics throughout the five cycles.

The repetitive fluorescence switching property leveraged the AIE mechanism inherent in the CN-TFPA dye within the confined space of the polyester fiber. At 25 °C, CN-TFPA dye remained in an aggregated state within the fibers, emitting strong fluorescence due to constraints on intramolecular motion. However, as the temperature increased, the motion of aggregated CN-TFPA molecules became more active, and the inner space of the fiber expanded, creating sufficient space for the formation of the isolated state of the CN-TFPA dyes. This temperature-induced expansion facilitated freer intramolecular rotation of the dyes, constraining radiative relaxation and ultimately resulting in a decrease in PL intensity [33,34,35]. 

**Figure 9:**
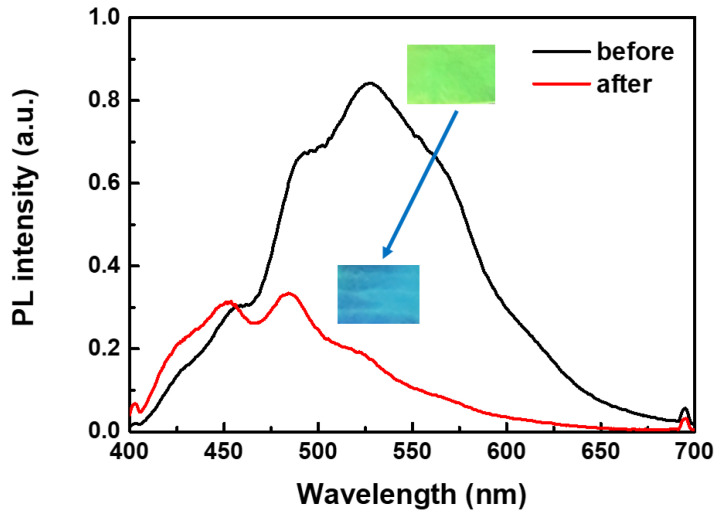
PL spectrum changes in AIE-dyed fabrics before and after exposure to DMF vapor. The inset images are photos of the fabric before and after DMF vapor treatment.

The aggregation of CN-TFPA dye could be controlled with external stimuli, such as solvent vapors, providing means for fluorescence on/off switching (Figure 9). Fabrics dyed with CN-TFPA emitted yellow fluorescence under a 365 nm illumination. However, upon exposure to DMF vapor, the solvent vapor penetrated between the CN-TFPA aggregates, locally weakening the molecular interactions and causing the rapid extinction of yellow fluorescence [36,37]. This on/off characteristic was also observed upon exposure to the other solvents, including THF, chloroform, and toluene vapors. When contact with organic solvent vapors was eliminated, the yellow fluorescence of the fabrics was reversibly restored. These results confirmed that the CN-TFPA dye could serve as a smart sensor capable of detecting changes in various external environments, extending its functionality beyond merely dyeing fabrics for fashion [38,39,40,41,42,43].

### 3.5. Mechanical Properties of AIE-Dyed Fabrics

**Figure 10:**
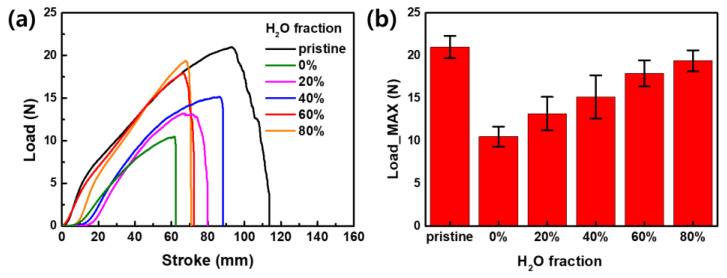
(**a**) Tensile test results and (**b**) maximum load of AIE-dyed fabrics according to the H_2_O fraction of the dye solution from 0 to 80%.

After the dyeing process using an organic solvent other than H_2_O, the decrease in strength of the dyed fabrics was evaluated through a tensile test. Figure 10 presents the strength measurement results of the dyed fabrics depending on the H_2_O fraction (0 to 80%) of the solution used for dyeing [44]. “Pristine” refers to a fabric sample that has been purified but not dyed. As anticipated, the fabric dyed with the pure DMF solvent without the use of H_2_O withstood the lowest load of 10.5 N. Furthermore, the fabrics dyed with a solution containing a 20% H_2_O fraction recorded a load more than 50% lower compared to that of the untreated fabrics. This decrease was attributed to the diminished interactions among polymer chains within the fibers induced by DMF, resulting in the weakened mechanical strength of the fabrics. However, as the H_2_O fraction in the dyeing solution increased, the load of the fabrics was gradually enhanced, and the 60% dyed sample, which showed the best fluorescence properties (Figure 5), demonstrated a reasonable strength of 17.9 N. The mixed solvent dyeing methods, employing DMF and high temperatures without surfactants, unavoidably led to a reduction in the strength of the fabrics. To mitigate these adverse effects, additional processing is required when contemplating the practical application of AIE-dyed fabrics [45]. 

## 4. Conclusions

In this study, the CN-TFPA dye with AIE properties was successfully incorporated into the recycled polyester fabric employing the mixed solvents system. First, the H_2_O fraction in the DMF solvent was systematically adjusted to assess the agglomeration of the dye particles and the resulting fluorescence emission characteristics. Additionally, the dyeing condition and fluorescence properties of the CN-TFPA dye on the recycled fabric were evaluated under the same conditions. As a result, the highest PL intensity was recorded at the H_2_O fraction of 60% in both suspension- and fabric-coating states. The AIE-dyed fabrics exhibited responsive thermochromic and vaporchromic properties, serving as potential smart sensors. As a thermochromic sensor, the PL intensity of the AIE-dyed fabric exhibited a consistent decrease with the rise in temperature. Finally, at 200 °C, the PL intensity was approximately 80% lower than that at room temperature. In addition, the AIE-dyed fabric also displayed the rapid extinction of yellow fluorescence emission under 365 nm illumination upon exposure to DMF vapor. However, the adoption of mixed solvent dyeing techniques involving the utilization of DMF and elevated temperatures inevitably engendered a reduction in the mechanical strength of the fabrics. In future research, we plan to enhance the mechanical strength of the AIE-dyed fabrics by incorporating reinforcing materials or implementing post-treatment processes. In addition, since synthetic AIE dye was used rather than a commercial dye, large amounts of solvent and long dyeing time were employed to overcome the low dispersibility and dyeing properties of AIE dye. Since this dyeing process is not desirable for environmental sustainability, we also planned to modify the molecular structure of the AIE dye to improve dispersibility and dyeing efficiency in the next study. Further improvements, including the enhancement of the mechanical strength and integration with advanced sensing technologies, could position AIE-dyed fabrics as promising candidates for smart textile sensors.

## Data Availability

Data are contained within the article.
