# Correction: Hong et al. Textile-Based Adsorption Sensor via Mixed Solvent Dyeing with Aggregation-Induced Emission Dyes. *Materials* 2024, *17*, 1745

**DOI:** 10.3390/ma17143609

**Published:** 2024-07-22

**Authors:** Seong Gyun Hong, Byeong M. Oh, Jong H. Kim, Jea Uk Lee

**Affiliations:** 1 Department of Advanced Materials Engineering for Information and Electronics, Integrated Education Institute for Frontier Science and Technology (BK21 Four), Kyung Hee University, 1732 De-ogyeong-daero, Giheung-gu, Yongin-si 17104, Gyeonggi-do, Republic of Korea; tjdrbs828@khu.ac.kr; 2 Department of Molecular Science and Technology, Ajou University, 206, World Cup-ro, Yeongtong-gu, Suwon-si 16499, Gyeonggi-do, Republic of Korea; hanmir980@ajou.ac.kr (B.M.O.); jonghkim@ajou.ac.kr (J.H.K.)

In the original publication [1], there was a small mistake in the molecular structure of Figure 1 and Figure 2, so it was corrected. The original figures will now be replaced by the following Figure 1 and Figure 2. The authors state that the scientific conclusions are unaffected. This correction was approved by the Academic Editor. The original publication has also been updated.

## Figures and Tables

**Figure 1:**
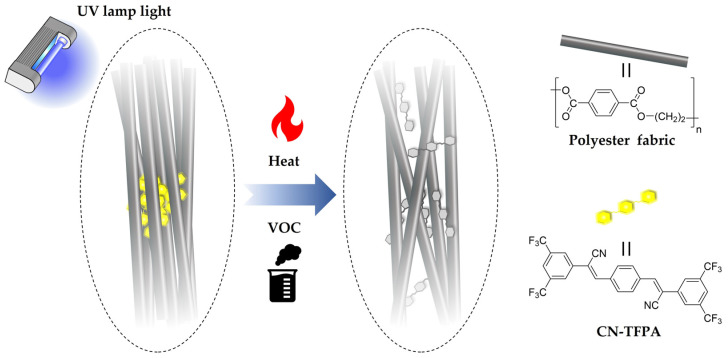
Schematic diagram of the sensing mechanism of AIE-dyed fabric.

**Figure 2:**
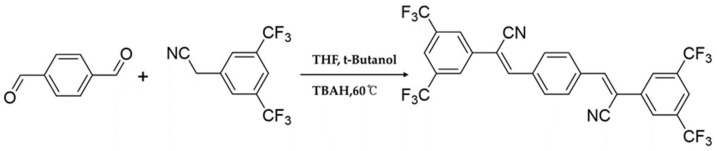
Synthetic procedure of CN-TFPA.

## References

[1] Hong S.G., Oh B.M., Kim J.H., Lee J.U. (2024). Textile-Based Adsorption Sensor via Mixed Solvent Dyeing with Aggregation-Induced Emission Dyes. Materials.

